# Low-Dose Cadmium Upregulates VEGF Expression in Lung Adenocarcinoma Cells

**DOI:** 10.3390/ijerph120910508

**Published:** 2015-08-28

**Authors:** Fuhong Liu, Bei Wang, Liqun Li, Fengyun Dong, Xiaocui Chen, Yan Li, Xiuzhen Dong, Youichiro Wada, Carolyn M. Kapron, Ju Liu

**Affiliations:** 1 Medical Research Center, Shandong Provincial Qianfoshan Hospital, Shandong University, 16766 Jingshi Road, Jinan 250014, Shandong, China; E-Mails: lfhmm2000@163.com (F.L.); liiqun99@163.com (L.L.); dong.fengyun@163.com (F.D.); chenxiaocui2015@sina.com (X.C.); 2 Department of Geriatric Medicine, Shandong Provincial Qianfoshan Hospital, Shandong University, 16766 Jingshi Road, Jinan 250014, Shandong, China; E-Mail: wangbei1224@126.com; 3 Children’s Health Center, Shandong Provincial Qianfoshan Hospital, Shandong University, 16766 Jingshi Road, Jinan 250014, Shandong, China; 4 School of Biomedical Engineering, Fourth Military Medical University, Changle West Road No. 169, Xi’an 710032, Shanxi, China; E-Mail: dongxiuzhen@fmmu.edu.cn; 5 The Research Center for Advanced Science and Technology, and Isotope Science Center, The University of Tokyo, 2-11-16, Yayoi, Bunkyo-ku, Tokyo 113-0032, Japan; E-Mail: wada-y@lsbm.org; 6 Department of Biology, Trent University, Peterborough, Ontario K9J 7B8, Canada; E-Mail: ckapron@trentu.ca

**Keywords:** cadmium, VEGF, lung adenocarcinoma, endothelial cell, angiogenesis

## Abstract

Cadmium (Cd) is a heavy metal and environmental toxin. Exposure to Cd has been associated with a variety of human cancers. In this study, we performed *in vitro* assays to examine the effects of cadmium chloride (CdCl_2_) on A549 cells, a human lung adenocarcinoma cell line. Cd does not affect proliferation, migration, or apoptosis of A549 cells at concentrations of 0.1–10 μM. At 0.5 and 1 μM, Cd increases the expression of vascular endothelial growth factor (VEGF) (*p* < 0.05, *p* < 0.01, respectively), but not basic fibroblast growth factor (b-FGF) in A549 cells. The conditioned media were collected from the A549 cells treated with 1 μM Cd and were co-cultured with human umbilical vein endothelial cells (HUVECs). Upon treatment with the conditioned media, the proliferation and migration of HUVECs significantly increased (*p* < 0.01, *p* < 0.05, respectively), while apoptosis remained unchanged. In addition, 1 μM Cd increases the level of hypoxia inducible factor 1-α (HIF1-α), which is a positive regulator of VEGF expression. Although low-dose Cd does not directly affect the growth of lung adenocarcinoma cells, it might facilitate the development of tumors through its pro-angiogenic effects.

## 1. Introduction

Lung cancer is a leading cause of cancer deaths worldwide [1]. Adenocarcinoma, arising from the epithelial cells of the small bronchi or bronchioles and typically peripherally located, is one of the four major histological types of lung cancer and has a wide spectrum of clinical and molecular features [2]. Over the past few decades, the incidence of adenocarcinoma has increased to be the most common type of lung cancer, accounting for about 50% of non-small cell lung cancers (NSCLC) [3]. Due to its unknown developmental mechanism, early metastasis, and insensitivity to radiation and chemotherapy, the five-year survival rate of lung adenocarcinoma is low [4]. 

Cadmium (Cd) is a toxic heavy metal that is widely used in industries that produce batteries and fertilizers [5]. It was classified as a carcinogen by the International Agency for Research on Cancer in 1993 [6]. Diet, cigarette smoking, and occupational exposure are the main sources of Cd exposure in humans [5,7]. Once absorbed, Cd travels in the body through the blood circulation, then distributes all over the body before accumulating in the liver and kidney [8,9]. The mechanisms of Cd-induced carcinogenesis has been demonstrated to involve multiple processes including aberrant gene expression, inhibition of DNA damage repair, induction of oxidative stress, and inhibition of apoptosis, depending on the dose, route, and duration of exposure [10,11,12]. A correlation between Cd exposure and adenocarcinoma is found in rodent inhalation studies, which demonstrate that a variety of Cd compounds produce dose-dependent increases in pulmonary adenocarcinomas [6]. *In vitro*, Cd induces inflammatory and proliferative responses in lung adenocarcinoma cells [13]. Since Cd has been observed to have biphasic effects depending on the experimental settings, it is not clear how Cd might affect the progression of lung adenocarcinoma. 

Angiogenesis, the formation of new blood vessels from the preexisting vasculature, plays an essential role in the growth, invasion, and metastasis of solid tumors [14,15]. Under hypoxic conditions, tumor cells can secrete several angiogenic factors such as vascular endothelial growth factor (VEGF) and basic fibroblast growth factor (bFGF) into the tumor microenvironment [16]. These growth factors bind to their receptors on endothelial cells, including VEGF receptor 2 and FGF receptor 1, and promote endothelial cell proliferation, migration, and survival [17]. The effect of Cd on tumor angiogenesis is still controversial. Vascular endothelium could be a primary target of Cd toxicity [9,18,19,20]. In addition, Cd impairs the capability of human breast cancer cells to induce angiogenesis [21]. However, Cd at concentrations of 5 and 10 μM increases VEGFR2 activity and tube formation in endothelial cells [22]. To date, little is known about the effects of Cd on the angiogenic potential of lung adenocarcinoma cells. 

In this study, A549 cells, a human lung adenocarcinoma cell line, were treated with a range of concentrations of Cd, and assayed for proliferation, migration, and apoptosis. Cd-induced expression and secretion of VEGF and b-FGF were also examined. The conditioned media derived from A549 cells treated with Cd were collected and co-cultured with human umbilical vein endothelial cells (HUVECs), which were later examined for angiogenic activities. We found that 1 μM Cd specifically upregulates VEGF expression and secretion in A549 cells, which consequently increases HUVEC proliferation and migration. In addition, 1 μM Cd increases hypoxia inducible factor 1-α (HIF1-α) in A549 cells. This study provides insights for understanding the effects of low-dose Cd on the development of lung adenocarcinoma. 

## 2. Experimental Section

### 2.1. Cell Culture

The human lung adenocarcinoma A549 cell line was purchased from Cell Resource Center of Life Sciences (Shanghai, China) and cultured in an RPMI 1640 medium (Corning Inc., Corning, NY, USA) containing 10% FBS, 100 U/mL penicillin, and 100 μg/mL streptomycin. HUVECs were purchased from American Type Culture Collection (Manassas, VA, USA) and cultured in EBM-2 medium supplemented with EGM-2 Single Quotes (Lonza, Walkersville, MD, USA) with 100 U/mL penicillin and 100 μg/mL streptomycin. All the cells were maintained in a humidified atmosphere of 5% CO_2_ at 37 °C and passaged twice a week by treating with 0.25% trypsin-EDTA (Life technologies, Gibco, CA, USA). CdCl_2_ was purchased from Sigma Aldrich (St. Louis, MO, USA). 

### 2.2. Cell Proliferation Assay

A549 cells’ and HUVECs’ proliferation was evaluated by an MTT assay kit (Cayman Chemical Company, Ann Arbor, MI, USA) following the manufacturer’s recommended protocol. Briefly, cells were seeded at a density of 5 × 10^3^ cells/well in a 96-well plate and cultured overnight. After treatment with CdCl_2_ or conditioned media, cells were washed with PBS and then MTT solution (10 μL of 5 mg/mL) was added to each well for 4 h. After the addition of 100 μL of Crystal Dissolving Solution, the formazan crystals were solubilized and the colorimetric intensity was analyzed using a 96-well plate reader (Molecular Devices, Sunnyvale, CA, USA) at a wavelength of 570 nm. Each experiment was repeated four times. 

### 2.3. Wound-Healing Assay

A wound-healing assay was used to assess the migration process [23]. A549 cells were seeded at a density of 3 × 10^5^ cells/mL in six-well flat-bottom plates and allowed to adhere overnight. At 90% confluence, wounds were made using a 10-μL pipette tip and the wells were washed twice with PBS to remove cellular debris. Then fresh medium with different concentrations of CdCl_2_ (0, 0.1, 0.5, 1, 5, and 10 μM) was added, and photographs were taken immediately (time zero) through an inverted microscope (Leica, Wetzlar, Germany). A549 cells were allowed to migrate for 12 h and photographed again. The experiments were carried out four times. Measurements were performed on digital images using the ImageJ software (NIH, Bethesda, MD, USA). At least 10 images per treatment were analyzed.

### 2.4. Annexin V-FITC/PI Analyses

Apoptosis of A549 cells and HUVECs was detected by Annexin V-FITC and propidium iodide (PI) staining using an assay kit (Neobiosciences, Shenzhen, China) according to the manufacturer’s protocol. Briefly, cells were pelleted and washed twice with PBS. Then, 1 × 10^6^ cells were resuspended in binding buffer. The single cells were stained with Annexin V-FITC (0.025%) for 3 min and PI (20 μg/mL) for 10 min in the dark. Detection of positive staining cells was performed using a FACS Aria^TM^ II flow cytometer (BD Biosciences, San Jose, CA, USA). The data were analyzed by the FACSDiva acquisition and analysis software. 

### 2.5. Quantitative Real-Time PCR (qRT-PCR)

A549 cells were collected after CdCl_2_ treatment by Trizol (Invitrogen, Carlsbad, CA, USA). RNA isolation were performed using the Total RNA Kit I (OMEGA, Norcross, GA, USA) and cDNA synthesis was performed using the RevertAid First strand cDNA Synthesis kit (Thermo Fisher, Grand Island, NY, USA). qRT-PCR was performed using a ViiA7 Real-Time PCR System (Applied Biosystems, Waltham, MA, USA). Reaction conditions were: 95 °C for 5 min, 40 cycles of 95 °C for 10 s, and 60 °C for 32 s. All PCR reactions were repeated in triplicate. Relative expression was calculated using GAPDH as an endogenous internal control. The primer sequences are summarized in Table 1. 

**Table 1:** qRT-PCR primer sequences.

Gene	Sequence	Size (bp)	Tm (ºC)
*VEGFA*			
Sense	AAAGGGAAAGGGGCAAAAACGAA	110	60.5
Anti-sense	AGGAACATTTACACGTCTGCGG		
*b-FGF*			
Sense	AGCGACCCTCACATCAAG	106	61
Anti-sense	ATCTTCCATCTTCCTTCATAGC		
*GAPDH*			
Sense	TGATGACATCAAGAAGGTGGTGAAG	240	60
Anti-sense	TCCTTGGAGGCCATGTGGGCCAT		

Notes: All sequences are in the 5’ to 3’ orientation; bp: base pair; Tm: temperature.

### 2.6. Western Blotting

A549 cells were washed with PBS and lysed in ice-cold RIPA buffer (20 mMTris pH 7.5, 150 mMNaCl, 50 mMNaF, 1% NP40, 0.1% DOC, 0.1% SDS, 1 mM EDTA, 1 mM PMSF, 1 μg/mL leupeptin). Protein concentration was determined using the BCA assay (Bio-Rad, Hercules, CA, USA). Equal amounts of protein were separated by SDS-PAGE (10% polyacrylamide gel) and transferred on to a PVDF membrane. PVDF membranes were blocked with 2.5% BSA and incubated overnight with a primary antibody in PBS-T at 4 °C. Primary antibodies were rabbit anti-VEGF (1:1000; Abcam, Cambridge, MA, USA), anti-HIF-1α (1:250; Proteintech, Wuhan, China), anti-GAPDH (1:2000; Cell Signaling Technology, Beverly, MA, USA) and anti-β-actin (1:6000; Proteintech). Immunoreactivity was visualized with HRP-conjugated secondary antibodies and chemiluminescence (ECL kit, Santa Cruz Biotechnology, Santa Cruz, CA, USA). Densitometry analysis was performed with ImageJ software (NIH).

### 2.7. ELISA

A549 cells were grown into confluence and applied with serum/growth factor-free media containing 1 μM CdCl_2_. After 12 h exposure, the media were collected for detection of VEGF or bFGF protein level using human VEGF Quantikine ELISA kit or human FGF basic Quantikine ELISA (R&D systems, Minneapolis, MN) following the manufacturer’s protocol. The measurements were performed four times (*n* = 4). 

### 2.8. Preparation of Tumor-Conditioned Medium (CM)

A549 cells were seeded at a density of 5 × 10^5^ cells/ml in a 65-mm dish with 1640 medium containing 10% FBS overnight. The medium was replaced with serum-free EMB2 medium with/without 1 μM CdCl_2_ and the cells were incubated for 12 h. The CM was collected and filtered with a 0.2-μm filter. The aliquots were stored at −80 °C in a freezer.

### 2.9. Electric Cell-Substrate Impedance Sensing (ECIS) Analysis

A real-time wound healing assay was performed using the ECIS technique (ECIS model 1600; Applied Biophysics, Troy, NY, USA) [24]. Briefly, eight-well ECIS arrays (8W10E+) were first coated with fibronectin (Invitrogen). Then, HUVECs were plated at a density that would allow for formation of confluent mono-layers directly on top of the electrodes. After treatment with control and Cd-conditioned media, AC current was given to the electrodes and the cells on the electrodes were killed. The viable cells surrounding the electrodes migrated into the wounded areas and the migration was measured by recording the trans-endothelial electrical resistance (TEER). Data plots are representative of triplicate experiments.

### 2.10. Statistical Analysis

The data are expressed as the mean ± standard error. The difference between the two groups was evaluated using a Student’s *t*-test (two-tailed). All statistical analyses were performed using SPSS 17.0 statistical software (SPSS Inc., Chicago, IL, USA). A *p* value < 0.05 was considered significant. 

## 3. Results

### 3.1. Low Dose of Cd Has No Significant Effect on Proliferation, Migration, and Apoptosis of A549 Cells

**Figure 1:**
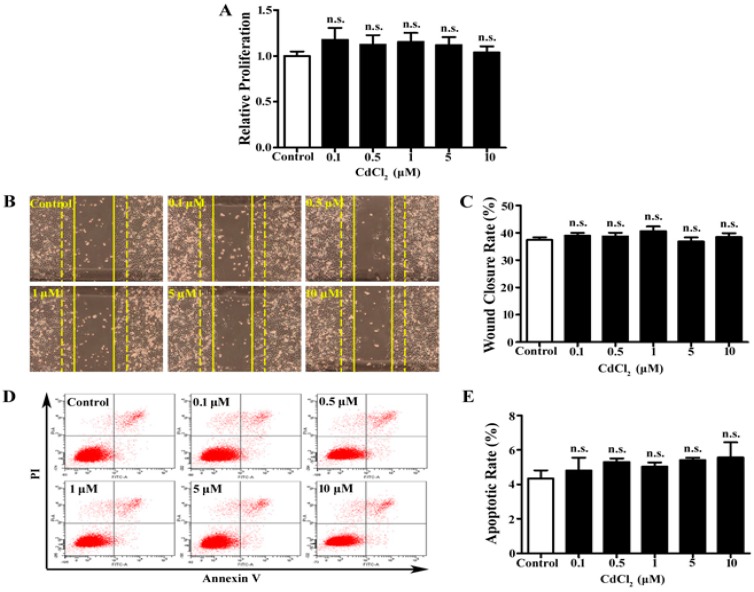
Proliferation, migration, and apoptosis of A549 cells with Cd treatment. (**A**) MTT assay of A549 cells treated with low concentrations (0–10 μM) of CdCl_2_. *n* = 6, n.s., non-significant. (**B**) Representative images of wound healing assay of A549 cells with Cd treatment. Dashed lines indicate 0 h and solid lines indicate 12 h. (**C**) Bar graph of wound closure rate of A549 cells. *n* = 10. (**D**) Representative image of flow cytometry with AnnexinV/PI double-staining for A549 cells treated with Cd. (**E**) Bar graph of A549 cell apoptotic rate following flow cytometry. *n* = 3.

Cd affects the cellular function of multiple types of cells depending on the dose and exposure time [25,26,27,28]. To examine the effects of Cd on A549 cells, CdCl_2_ at the concentrations of 0.1, 0.5, 1, 5, and 10 μM was applied to the cell culture. MTT assay was performed to determine cell proliferation. We found that 24-h Cd treatment did not significantly change A549 cells’ proliferation at all concentrations (Figure 1A). Cell migration was evaluated by a wound healing assay, and the wound closure rates were largely unchanged in all Cd-treated groups of A549 cells (Figure 1B, 1C). In addition, apoptosis was measured with Annexin V-FITC/PI double-labeled flow cytometry. The apoptotic rate was calculated as the percentage of the early and late apoptotic cells. As shown in Figure 1D and 1E, no significant change in the apoptotic rate was observed in Cd-treated A549 cells. Thus, up to 10 μM, Cd treatment does not affect A549 cell proliferation, migration, and apoptosis. 

### 3.2. Low-Dose Cd Upregulates VEGF but Not b-FGF Expression in A549 Cells

**Figure 2:**
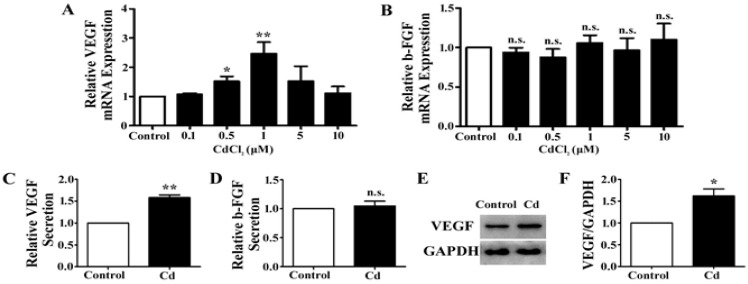
The effects of Cd on VEGF and b-FGF expression in A549 cells. (**A**) Relative VEGF mRNA expression in A549 cells treated with CdCl_2_ for 24 h by qRT-PCR. *n* = 4; n.s., non-significant; *, *p* < 0.05; **, *p* < 0.01. (**B**) Relative b-FGF mRNA expression in A549 cells treated with CdCl_2_ for 24 h by qRT-PCR. *n* = 4; n.s., non-significant. (**C**) Relative VEGF secretion of A549 cells treated with 1 μM Cd for 24 h. *n* = 4; **, *p* < 0.01. (**D**) Relative b-FGF secretion of A549 cells treated with 1 μM Cd for 24 h. *n* = 4; n.s., non-significant. (**E**) Representative immunoblot of VEGF and GAPDH in A549 cells treated with 1 μM Cd for 24 h. (**F**) Densitometry analyses of the blots of VEGF/GAPDH. *n* = 3;*, *p* < 0.05.

VEGF and b-FGF signaling are the fundamental regulators of angiogenesis [29]. We examined the expression level of VEGF and b-FGF mRNA in A549 cells treated with different doses of Cd. When cells were exposed to Cd concentrations of 0.5 μM and 1 μM, the expression of VEGF mRNA significantly increased (0.5 μM: 1.52 ± 0.17-fold, *p* < 0.05; 1 μM: 2.5 ± 0.39-fold, *p* < 0.01), whereas it is not significantly affected at 0.1 μM, 5 μM, and 10 μM of Cd treatment (0.1 μM: 1.07 ± 0.04-fold, *p* = 0.05; 5 μM: 1.53 ± 0.51-fold, *p* = 0.154; 10 μM: 1.11 ± 0.23-fold, *p* = 0.468) (Figure 2A). At all concentrations, Cd treatment did not significantly change the mRNA levels of b-FGF in A549 cells (Figure 2B). The concentration of 1 µM Cd was used for the later experiments as it induced the greatest increase of VEGF mRNA expression in A549 cells.

To examine the effect of Cd on VEGF and b-FGF secretion, A549 cells were treated with 1 µM Cd in serum free media for 12 h, and the media were collected for ELISA. We found that the level of VEGF protein in the media was significantly increased (1.58 ± 0.11-fold, *p* < 0.01) (Figure 2C), while the level of b-FGF protein remained unchanged (1.05 ± 0.14-fold, *p* = 0.74) (Figure 2D). In addition, the lysate of A549 cells treated with 1 µM Cd was collected for examination of the cellular protein level of VEGF by Western blotting. As shown in Figure 2E, VEGF protein is increased in Cd-treated cells and the increase was verified by densitometry analysis (Control: 1.11 ± 0.13; Cd: 1.79 ± 0.17, *p* < 0.05) (Figure 2F). These results indicate that1 µM Cd increases both the expression and secretion of VEGF in A549 cells.

### 3.3. Conditioned Media Derived from Cd-Treated A549 Cells Promotes Endothelial Cell Proliferation and Migration 

**Figure 3:**
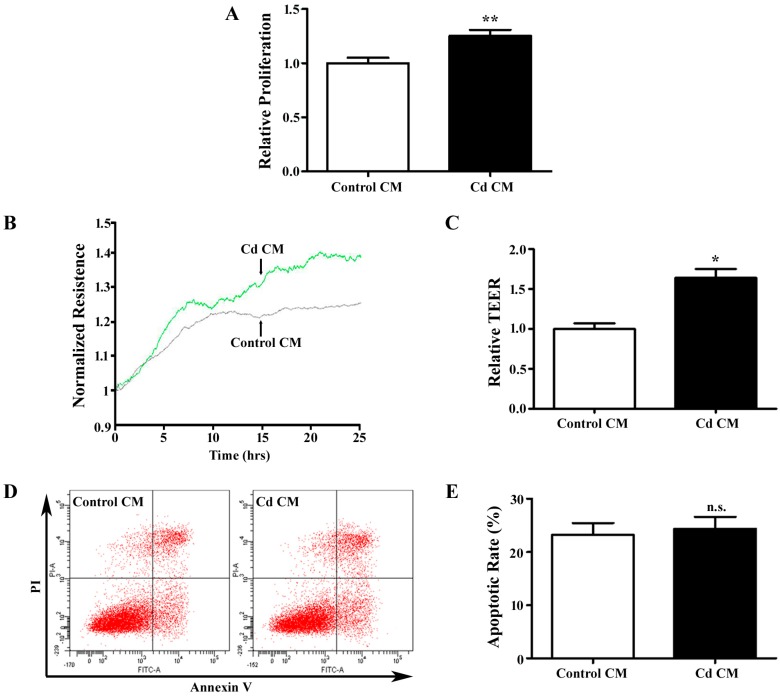
The effects of a Cd-treated A549 cells-derived conditioned medium (CM) on HUVECs. (**A**) MTT assay for HUVECs cells treated with Cd CM. *n* = 6; **, *p* <0.01. (**B**) Real-time transendothelial electrical resistance (TEER) measurement of HUVEC monolayer treated with Cd CM. (**C**) Bar graph of the mean percentage of TEER. *n* = 4; *, *p* < 0.05. (**D**) Representative image of flow cytometry with AnnexinV/PI double-staining for HUVECs treated with Cd CM. (**E**) Bar graph of apoptotic rate of HUVECs following flow cytometry. *n* = 3; n.s., non-significant.

Stimulation of endothelial cells by tumor cell-secreted angiogenic growth factors is an crucial step in angiogenesis [18]. Conditioned media (CM) from tumor cells has been widely used in *in vitro* angiogenesis assays to mimic *in vivo* angiogenesis events [30]. The confluent A549 cells were treated with 1 µM Cd in serum free media for 12 h, and the media were collected for co-culture with HUVECs. The CM from A549 cells without Cd treatment was used as a control. After being challenged with the Cd for 12 h, HUVECs were assessed using the MTT assay, which indicated that Cd CM significantly elevated HUVEC proliferation (1.25 ± 0.06, *p* < 0.01) (Figure 3A). To accurately evaluate the progress of cell migration, we employed ECIS system, which detects real-time resistance caused by endothelial cell migration. The transendothelial resistance of HUVECs was significantly increased in Cd CM (1.64 ± 0.19, *p* < 0.05), suggesting that cell migration is increased by secretions of A549 cells treated with Cd. We also examined apoptosis of HUVECs treated with conditioned media by Annexin V-FITC/PI double-labeled flow cytometry. As shown in Figure 3B and C，the apoptotic rates were similar in HUVECs treated with control and Cd CM (Control CM: 23.27 ± 2.17%; Cd CM: 24.37 ± 2.24%, *p* = 0.742). Taken together, Cd-treated A549 cell CM promotes proliferation and migration, but does not affect apoptosis of endothelial cells. 

### 3.4. Low-Dose Cd Increases HIF-1α in A549 Cells 

**Figure 4:**
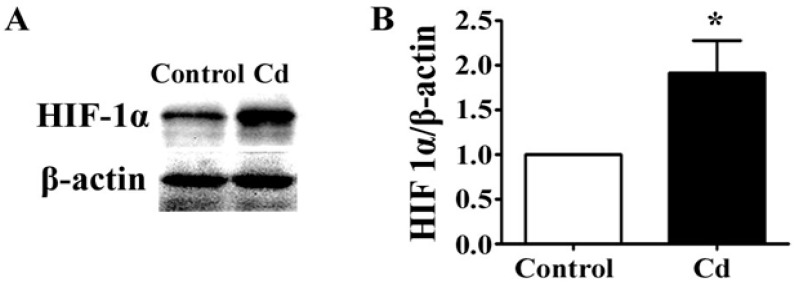
The effects of Cd on HIF-1α in A549 cells. (**A**) Representative immunoblot of HIF-1α and β-actin in 1 μM Cd-treated A549 cells for 24 h. (**B**) Densitometry analyses of the blots of HIF-1α/β-actin. *n* = 3; *, *p* < 0.05.

The expression of VEGF is activated by HIF-1, a transcription factor which regulates the cellular responses to hypoxia [31]. HIF-1 is a heterodimeric basic helix-loop-helix protein, which consists of a regulatory subunit, HIF-1α, increasing under hypoxic conditions, and a constitutively expressed subunit, HIF-1β [32]. By Western blotting, we found that 1 μM Cd significantly elevated the level of HIF-α in A549 cells (Figure 4A,B, *p* < 0.05). Thus, low-dose Cd might upregulate VEGF expression by accumulating HIF-1α protein in A549 cells. 

## 4. Discussion

Cd exposure causes a series of severe clinical symptoms and has been proven to be a lung carcinogen [5]. However, its effects on the progression of lung adenocarcinoma are not clear. In this study, we found that Cd does not affect A549 cell proliferation, migration, and apoptosis at concentrations up to 10 μM. In addition, low-dose Cd upregulates the expression and secretion of the angiogenic factor VEGF, but not b-FGF. The conditioned media from Cd-treated A549 cells stimulates endothelial proliferation and migration. Thus, low-dose Cd might facilitate the development of lung adenocarcinoma through its pro-angiogenesis effects. 

Cd is not biodegradable and persists in the body after intake. Prolonged exposure to Cd causes cytotoxity due to its accumulation in a variety of cell types over time. Short-term Cd exposure affects cell cycle, proliferation, differentiation, DNA replication, and repair, as well as apoptotic pathways. In epithelial cells, Cd alters the cellular homeostasis of secondary messengers, such as reactive oxygen species (ROS) and Ca^2+^. Cd also activates gene expression of c-myc and c-Jun, and inhibits tumor suppressor genes such as p53 and p27 [33,34]. Depending on the experimental settings, Cd usually induces disruption of cellular functions at a higher dose or longer exposure time, e.g., Cd upregulates the proliferative responses of A549 cells after 72 h of exposure [13]. In the human body, short-term and low-dose exposure to Cd is more common. Our study showed no significant effects on cell proliferation, migration, and apoptosis when A549 cells were exposed to 0 to 10 μM Cd for 24 h. This suggests that a low dose of Cd might not have direct effects on the growth of lung adenocarcinoma. 

The rapid proliferation and metastatic nature of tumor cells rely upon support from tumor blood vessels in the form of angiogenesis [35]. VEGF plays a critical role in angiogenesis, and has been found to be expressed in various human lung cancers including lung adenocarcinoma [36]. By increasing vascular permeability, VEGF allows leakage of multiple plasma proteins, some of which degrade the extracellular matrix to create space for cell growth and migration [37]. VEGF is also a potent mitogen for endothelial cells, which proliferate and migrate into the tumor to form new capillaries [38]. In addition, VEGF serves as a survival factor and inhibits apoptosis of endothelial cells of the newly formed vasculature [39]. Basic FGF is another pro-angiogenic factor that potently stimulates endothelial cell proliferation [40]. A549 cells secrete VEGF and b-FGF [41]. In this study, we found that a low dose of Cd specifically upregulates expression and secretion of VEGF. Expression of VEGF is regulated by a collection of transcription factors, one of which is HIF-1α [31]. Cd inhibits HIF-1α activities in several cell types; however, it elevates HIF-1α expression through ROS, ERK, and AKT signaling pathways in human bronchial epithelial cells [8]. Our study demonstrates that low-dose Cd increases the protein level of HIF-1α. Thus, HIF-1α might mediate Cd-induced upregulation of VEGF. 

At the beginning of angiogenesis, VEGF binds to its receptor VEGFR2 on the membrane of endothelial cells [17]. VEGFR2 is phosphorylated and activates the downstream intracellular pathways that promote endothelial cell proliferation, migration, and survival [42]. In our study, we found that conditioned media collected from Cd-treated A549 cells promotes proliferation and migration of HUVECs, which might be the result of a higher level of VEGF and/or other ingredients in the conditioned medium. The conditioned medium did not inhibit endothelial cell apoptosis, possibly due to the relatively short co-culture time. The conditioned medium contains Cd, which may have an effect on endothelial cells by direct interaction. However, previous studies indicated that Cd induces cytotoxicity on HUVECs only at a concentration above 10 μM [43]. We have also reported that 4 μM Cd does not alter HUVEC growth and viability up to 48 h [9]. Therefore, the altered growth and migration of endothelial cells were likely induced by segregates from A549 cells, not by Cd itself. 

In summary, we demonstrate that a low dose of Cd specifically upregulates VEGF expression, which might subsequently promote endothelial cell proliferation and migration. Our study suggests that low-dose exposure to Cd might facilitate the growth of lung adenocarcinoma by promotion of angiogenesis, and thus provides information important to environmental regulations and occupational protection, as well as chemotherapy of human lung adenocarcinoma. 

## 5. Conclusions

Low-dose Cd does not affect proliferation, migration, or apoptosis of lung adenocarcinoma cells, but specifically upregulates the expression and secretion of VEGF. Although low-dose Cd has no direct effects on the growth of lung adenocarcinoma cells, it might facilitate the development of tumors through its pro-angiogenic effects.
